# The Role of Supplementary Cementitious Materials (SCMs) in Ultra High Performance Concrete (UHPC): A Review

**DOI:** 10.3390/ma14061472

**Published:** 2021-03-17

**Authors:** Sungwoo Park, Siyu Wu, Zhichao Liu, Sukhoon Pyo

**Affiliations:** 1 Department of Urban and Environmental Engineering, Ulsan National Institute of Science and Technology (UNIST), Ulsan 44919, Korea; paksungwoo@unist.ac.kr (S.P.); wsy86023@unist.ac.kr (S.W.); 2 State Key Laboratory of Silicate Materials for Architectures, Wuhan University of Technology, Wuhan 430070, China; liuzc9@whut.edu.cn; 3 School of Materials Science and Engineering, Wuhan University of Technology, Wuhan 430070, China

**Keywords:** ultra high-performance concrete (UHPC), supplementary cementitious materials (SCMs), sustainability, compressive strength, flowability, shrinkage

## Abstract

Although ultra high-performance concrete (UHPC) has great performance in strength and durability, it has a disadvantage in the environmental aspect; it contains a large amount of cement that is responsible for a high amount of CO_2_ emissions from UHPC. Supplementary cementitious materials (SCMs), industrial by-products or naturally occurring materials can help relieve the environmental burden by reducing the amount of cement in UHPC. This paper reviews the effect of SCMs on the properties of UHPC in the aspects of material properties and environmental impacts. It was found that various kinds of SCMs have been used in UHPC in the literature and they can be classified as slag, fly ash, limestone powder, metakaolin, and others. The effects of each SCM are discussed mainly on the early age compressive strength, the late age compressive strength, the workability, and the shrinkage of UHPC. It can be concluded that various forms of SCMs were successfully applied to UHPC possessing the material requirement of UHPC such as compressive strength. Finally, the analysis on the environmental impact of the UHPC mix designs with the SCMs is provided using embodied CO_2_ generated during the material production.

## 1. Introduction

Ultra high-performance concrete (UHPC) is one of the leading construction materials with greatly advanced properties compared to conventional concrete. A cementitious mixture of which the compressive strength is over 120 MPa belongs to the UHPC category according to ASTM C1856 [1], while the ACI committee reported that the compressive strength of UHPC should be greater than 150 MPa [2,3,4]. In addition to the remarkable compressive strength, UHPC, designed based on a particle packing theory, also possesses superior durability compared to conventional concrete with the help of dense microstructure [5,6,7,8,9,10,11,12,13,14,15]. For example, it has strong resistance to water permeability, chloride penetration, freeze and thaw, and chemical attack because of its great material properties. Comprehensive studies on UHPC have been conducted for the last few decades such as the rheological properties of a fresh paste of UHPC [15,16,17,18,19,20], the effect of fibers [16,19,21,22,23], mix design [24,25,26,27,28,29], and structural applications [30,31,32,33].

Perhaps, the disadvantage of UHPC comes from the standard ingredient, specifically, a large portion of Portland cement. The cement industry is well known to generate 8–9% of global CO_2_ emissions [34]. Even though the structure member size can be smaller with UHPC than conventional concrete because of its high strength [35], UHPC generally contains cement about three times higher than normal concrete by volume [25,36]. Furthermore, not all cement particles in UHPC are hydrated in an extremely low w/c ratio environment. The hydration degree is reported as only 52–61% with a w/c ratio of 0.23–0.33 [37] and the unhydrated cement makes UHPC not eco-friendly [12,38]. Therefore, the possible approach to reduce cement content in UHPC is replacing part of the cement with supplementary cementitious materials (SCMs) such as slag, fly ash (FA), limestone powder (LP), metakaolin (MK), and other SCMs. Most SCMs are industrial by-products or naturally occurring materials: slags are by-products in the production of iron, steel, lithium carbonate, phosphor, or copper; FA is a by-product of a coal power plant; and SF is a by-product from the production of elemental silicon or alloys with silicon. In addition, limestone is a natural occurring material, and MK is obtained from the calcination of kaolinite.

This paper reviewed the effect of various SCMs on UHPC performances. SCMs can be categorized into slag, FA, LP, MK, and other SCMs. The effects of SCMs on compressive strength, flowability, porosity, shrinkage, and environmental impacts of UHPC are discussed. The paper also introduces the method of how to evaluate the environmental effect of each SCM. The objective of the paper is to summarize the current state of SCM application and its performances in UHPC.

## 2. Summary of SCMs Reviewed

The SCMs reviewed in this paper are summarized in Table 1. The table shows the types of SCMs that have been studied in the literature and their effects on the UHPC’s performances. The performances of UHPC investigated are early (at 3 days or earlier) and late compressive strength (later than 3 days), flowability, porosity, and shrinkage. The effects of SCMs were compared to the references in each study. For example, high “Early compressive strength” means that usage of SCMs exhibits higher early compressive strength than a reference mix design. The numbers in the column of “SMC No.” are linked to the numbers in the column of “List of SCMs” and the information of SCMs or a combination of SCMs can be found.

When a combination of SCMs is used, the effects of the main SCM are reported. Many studies investigated the usage of combinations of SCMs and it is inefficient to regard each combination as a single different SCM. Therefore, the authors picked one representative SCM in a combination and investigated its effect. The representative SCM in a combination is bolded in the table and others are referred to as a reference. For example, (8) in the “List of SCMs” column in Table 1 used three SCMs: **GGBS (25.5%)** + SF + BA. The compressive strength with the different dosages of GGBS was compared and discussed in the slag section (see Section 3.1). The UHPC with GGBS exhibits lower early age compressive strength compared to the reference with the combination of SF and BA only. In the case of italic texts for the SCMs, the reference is different from the mix designs with SCMs and is not able to be written in the Table 1 because of space limitations. For example, (22) in the “List of SCMs” column in Table 1 compared the effect of *“FFA (20%) + MK(3.8%)*” with the UHPC only with SF. In this case, it is recommended to find more detailed information on the reference mix design through the papers cited. Lastly, the replacement ratio is the weight of the SCM to total solid binder weight, which resulted in the highest compressive strength. For example, (8) in the “List of SCMs” column in Table 1 GGBS is used along with BA and SF. The replacement ratio of 25.5% is obtained from dividing GBBS weight by the sum of cement, GBBS, BA, and SF.

Various combinations of SCMs in UHPC mix designs have been studied by many research groups. Most UHPC studies used SF as a main ingredient and parts of the cement were replaced with other SCMs in the literature. Although there are various combinations, most SCMs are placed in the ordinary categories: slag, FA, LP, and MK. Some other SCMs such as RHA, NP, NMC, DCP, CKD, GGP, BP and FGP are also investigated. The effects of the SCMs on the UHPC performances of early and late compressive strength, flowability, shrinkage, and the environmental impact are discussed comprehensively in the following sections.

## 3. Effect of SCMs on Material Properties of UHPC

### 3.1. Slag

Slag is the by-product in pig iron, steel, lithium carbonate, phosphor, or copper plants and their primary oxide components are CaO, SiO_2_, and Al_2_O_3_, although the proportions are not the same among different sources [72]. The effects of slag on UHPC compressive strength, flowability, and shrinkage are summarized in Table 2, Table 3 and Table 4, respectively. They also include information such as the water to binder (w/b) ratio, the curing method, specimen size, the superplasticizer to binder (SP/b) ratio, the aggregate to binder (Agg/b) ratio, the mixture type, and other solid ingredients. The same types of tables are used for other SCMs in the following subsections.

Slag tends to decrease the compressive strength of UHPC at an early age because of its low reactivity. Slag has hydraulic properties and reacts with water [73] and the hydration product of slag is calcium silicate hydrate (CSH) [43,74]. Slag is chemically activated by calcium hydroxide (Ca(OH)_2_) and gypsum in cement, but its reaction speed is slow, whereas SF reacts with Ca(OH)_2_ first in UHPC because of its fineness [44,75]. Researchers have also reported that slag decreases the heat of hydration [39,43]. As a result, slag tends to decrease the compressive strength of UHPC at 3 days or earlier. Most of the research revealed that the compressive strengths at 3 days of the UHPCs with slag decrease by around 4.8–18.1% from the reference specimens. Pyo and Kim revealed that the addition of GGBS decreases the compressive strength at 1 day and 3 days by 39% and 18%, respectively [40]. They claimed that GGBS slow down the hydration process and setting time, which causes low early strength. Li et al. reported that SSP improves the workability even though it degrades the mechanical properties and durability of UHPC [12]. The 15% replacement of cement with SSP results in 8.7% lower compressive strength at 1 day than that of the reference specimen. As the SSP has a low hydration activity as well as a retarding effect on the cement hydration, the activity of SSP is lower than OPC and it slowed down the early age hydration. He et al. replaced SF with the LTS that is a by-product in the process of lithium carbonate [50]. The 10% of the LTS to the total binder results in a 4.8% lower compressive strength at 3 days compared to the control mix with SF only. The addition of LTS decreases the early age compressive strength because the pozzolanic reaction of LTS is slower than SF.

Slag could enhance the late age compressive strength of UHPC; the secondary pozzolanic reaction between slag and Ca(OH)_2_ in the pore solution produces additional CSH, which increases the packing density of the UHPC [44]. Liu et al. found that compressive strength increases up to 9% when GGBS content increased to 40% of the binder because the secondary pozzolanic reaction of GGBS is accompanied by consumption of Ca(OH)_2_ and the densification of the hardened paste [44]. Abdulkareem et al. reported that the use of GGBS can accelerate the hydration reaction of cement and also improve packing density because its fineness is between those of cement and SF [45]. When GGBS content increased to 23.6% of binder, the compressive strength increased by 3.3% at 7 days because of the improved packing density and higher cement hydration due to the addition of GGBS. Gupta used GGBS with 35% calcium oxide content that contributes to CSH formation, and thus, improves strength development [39]. The 60% replacement of cement with GGBS resulted in a 5.2% increase in the UHPC’s compressive strength at 28 days. Yu et al. reported a 10% increase in 28-day compressive strength by replacing 30% of the cement with GGBS [43]. He et al. successfully replaced 10% of cement with LTS and the compressive strength increases by 2.8% and 6.8% at 28 days and 90 days, respectively [50]. The fineness of LTS is between those of cement and SF, which can improve the packing density of UHPC and the pozzolanic reaction of LTS also contributes to the late age compressive strength development. Peng et al. used PS that has a similar glass structure to GGBS [48]. When PS content increased from 30% to 35%, the compressive strength increased by 3.7% because the addition of PS increased both the degree and speed of the pozzolanic reaction, which results in more hydration products in the paste and low porosity. The PS used by Yang et al. has relatively lower reactivity than ordinary slag because of the lower Al_2_O_3_ content [47]. P_2_O_5_ in the PS can also retard hydration at an early age. The PS of 27.4% of the binder was found to increase the compressive strength at 28 days by 6.3%. The PS can yield small pores in microstructure at an early age and fill those by the hydration product from the long-term pozzolanic reaction. Edwin et al. proved that CS can be used as an SCM in UHPC increasing the amount of CSH [51]. CS is a by-product of the copper metal smelting process. When CS content increases up to 16% of the binder, the compressive strength of UHPC increases by 3.1% at 90 days. Ahmadm et al. studied the effect of using industrial waste materials like PSS to replace parts of SF [49]. When replacing 20% of SF, the compressive strength of UHPC can still reach 161 MPa at 28 days, which is the same as that of the reference specimen with SF only and flow diameter slightly decreased, but it was still within the acceptable range.

The particle size of slag can be a critical factor in the compressive strength of UHPC. Randl et al. studied the effect of GGBS and FGGBS on the UHPC compressive strength [42]. The GGBS and FGGBS have the Blaine values of 4790 and 5620 cm^2^/g, respectively. When 38.5% of cement is replaced with FGGBS, it decreases the compressive strength by 1.5%, whereas GGBS decreases the strength by 16.1% because FGGBS results in a higher packing density than GGBS. The study concluded that the packing density of UPHC is an important factor even more than the hydraulic reactivity of slag. Pyo and Kim compared slags with different particle sizes [40]. The median particle size of FGGBS and GGBS are 2.69 μm and 14 μm, respectively. They reported that the usage of FGGBS increases the compressive strength increased by 7.4% at 3 days, while GGBS decreases the compressive strength with the same dosage of FGGBS. From the hydration heat measurement, they concluded that FGGBS plays a significant role in the early hydration process, which results in a higher compressive strength than the reference specimen.

Some slags may decrease the porosity of UHPC. Liu and Guo studied the effect of SSP on the compressive strength of UHPC [46]. The particle size distribution of SSP is similar to that of cement. It was found that the compressive strength decreased rapidly when the SSP content is high. With the 16.9% replacement ratio of cement with SSP, the compressive strength decreased by 10.3% compared to the reference specimen that contains SF only because SSP increases the proportion of the pores larger than 50 mm by 33%.

The effect of slag on the UHPC shrinkage possibly depends on the type of slag. Table 4 summarizes the effect of slag on the UHPC shrinkage. It has been proved that the addition of GGBS increases the shrinkage of conventional concrete because slag increases the self-desiccation by consuming pore solution (calcium hydroxide) in a small capillary pore structure [76,77,78]. However, different effects of different types of slags on the UPHC shrinkage have been observed in some studies in the literature. Li et al. found that the total shrinkage of UHPC incorporating SSP is lower than the UHPC without SSP [12]. As the amount of SSP increases, hydration of cementitious materials decreases at early ages, water consumption is reduced, and, thus, the self-desiccation of UHPC becomes weaker. Yang et al. indicated that as the cement replacement ratio with PS increases, the hydration can be slowed down and the cement dilution effect can improve the UHPC volume stability [47]. In other words, the high volume of PS can reduce the autogenous shrinkage of UHPC. On the other hand, Liu et al. found that the addition of GGBS can increase autogenous shrinkage [44]. They insisted that the secondary pozzolanic reaction of GGBS increases the consumption of calcium hydroxide, which increases water consumption. The higher water consumption results in more self-desiccation. They concluded that the secondary pozzolanic reaction of GGBS increases the autogenous shrinkage due to the refined pore structure and the increased depletion of water.

Slag increases the UHPC flowability because of its lower water absorption compared to cement having a slippery surface [79]. Table 3 summarizes the effect of slag on the UHPC flowability. Pyo and Kim reported that the addition of FGGBS and GGBS increases the UHPC flowability by 11.6% and 4.1%, respectively, compared to the reference specimen with SF only [40]. Yang et al. reported that the use of PS can significantly improve the flowability of UHPC. The flowability of UHPC can increase by 17.2% when the cement replacement ratio with PS increased up to 34.2% because it reduces the water absorption [47]. Furthermore, the addition of PS provides the cement dilution effect, which increases the water to cement ratio of UHPC with PS indirectly. Abdulkareem et al. reported that the workability is improved by the addition of GGBS [45]. In the paper, with the increase in GGBS, the dosage of superplasticizer should be reduced to achieve the same level of slump flow. Liu et al. used GGBS to improve the flowability of UHPC. With up to 30% replacement of cement with GGBS, the slump flow can increase by 6.3% because of the smooth surface and lower water absorption of the slag compared to cement [44]. Liu and Guo proved that the addition of SSP can improve the flowability of UHPC. The 16.9% replacement ratio of cement increases the flow diameter by 26.8% because the activity of SSP is lower than that of cement and the water requirement of SSP is also less than that of cement [46]. However, Li et al. found that the 15% replacement of cement with the different SSP source slightly decreases the flowability of UHPC because of the higher specific area than that of cement, which caused higher water demand [12]. Therefore, it should be pointed out that a higher specific area of different sources of slag could decrease the flowability of UHPC. Randl et al. found that the specific area of GGBS and FGGBS can improve the flowability of UHPC [42]. The slump flow of UHPC increased by 1.8% with 38.5% GGBS replacement and the slump flow of UHPC increased by 10.7% with 38.5% FGGBS replacement.

Slag is possibly more helpful than FA to improve the UHPC’s compressive strength. Wu et al. reported that when the cement replacement ratio is same, slag exhibits a higher compressive strength of UHPC than FA [41]. The 30% replacement ratio of cement with GGBS results in 5.7% and 4.0% lower at 3 days and 28 days, respectively, compared to the reference specimen containing SF only, while the 30% replacement with FA results in 13.5% and 8.0% lower at 3 days and 28 days, respectively.

Although slag is apt to decrease the early compressive strength of UHPC, many studies have demonstrated that it successfully improves the late compressive strength. The additional CSH produced by the secondary pozzolanic reaction between slag and Ca(OH)_2_ increases the density of the matrix and the compressive strength, which happens at the late age because of the slow hydration of slag. The disadvantage of the low early compressive strength is assumed to be overcome by adopting heat treatment as it increases the pozzolanic reactivity. The particle size of slag is also an important factor to increase the compressive strength of UHPC; slag of a finer particle size exhibits higher compressive strength. However, the extra grinding work increases the material cost and, therefore, finding reactive slag material seems a more efficient option. Slag decreases the water demand of UHPC because of its lower water absorption compared to cement. It is another critical factor to increase the compressive strength of UHPC because a lower w/c ratio can increase the compressive strength.

### 3.2. Fly Ash (FA)

FA is a by-product of power plants and is collected during the process of coal combustion. The chemical composition and particle size of FA are different from plant to plant, but it is generally a fine spherical powder, which increases the workability of conventional cementitious material. As a pozzolanic material, it is known that FA increases the late age strength of conventional cementitious material. The usage of FA can reduce CO_2_ emissions [55,66] and decrease the production cost and energy of concrete [41,49,53,55]. In recent years, many researchers have focused on developing new UHPC mixtures with locally available FA because substituting cement and/or SF with FA can reduce environmental impacts.

The effect of FA on UHPC compressive strength is summarized in Table 5. The compressive strength data reported show around 95 MPa at 3 days, 110–185 MPa at 28 days, and 152–202 MPa at 91 days with a 10–20% replacement of binder materials. It has been shown that the UHPC with FA exhibits a lower compressive strength than those of reference specimens. Ahmad et al. replaced a part of SF with FA, and found that using FA to substitute the SF to up to 11.8% of the binder slightly decreases the compressive strength by 1.9% compared to the reference specimen at 28 days [49]. Although FA degrades the compressive strength of UHPC, the value is higher than the minimum requirement of 150 MPa and the usage of FA can reduce the cost of UHPC. It has been proved that FA can improve many characteristics of high strength mortar. However, Pyo et al. found that replacing 12.8% cement with FA decreased the compressive strength by 48.9% and 6.1% at 1 day and 3 days, respectively, because of the high crystallinity of FA [40]. Wu et al. also investigated the effect of FA as an SCM for concrete, and concluded that the FA has negative effects on the compressive strength of UHPC [41]. The 15% replacement ratio of cement with FA results in 13.5% and 8% lower than those of reference specimen at 3 days and 28 days, respectively. Alsalman et al. adopted that FA can be used as an SCM for UHPC to reduce the cost of UHPC [53]. It was found that adding FA up to 15% of the binder significantly decreases the compressive strength by 33.7% at 1 day because the addition of FA delayed the strength development at early ages. The compressive strength of UHPC becomes similar to that of the reference specimen after the normal curing of 7 days or longer. Randl et al. found that the 38.5% replacement ratio of cement with FA decreases the compressive strength by 24.9% at 28 days compared to the reference specimen, even though the packing density is higher [42]. Therefore, it is inferred that the slow pozzolanic reaction of FA degrades the compressive strength of UPHC. It should be noted, however, that there are occasional studies reporting different trends. Šeps et al. replaced the cement of 30% with FA and it results in 19% higher compressive strength at 28 days than that of the reference specimen containing SF only [52].

Some of the FFA can improve the compressive strength of UHPC. Ferdosian and Camões introduced the method of how to optimize the UHPC mix design that satisfies the requirements of the compressive strength and the flowability using FFA of which the mean particle size is 4.48 μm [56]. They suggested the eco-efficient mix design that releases the lowest CO_2_ and the cost-efficient mix design that maximizes the amount of FFA and sand as well as minimizes the amount of SF. The eco-efficient mix design results in the compressive strength being 6.8% higher than the reference samples using the FFA of 34.1% in the binder.

The ternary use of SCMs including FA also can be a feasible solution to reduce the amount of cement and SF in UHPC. Li found that the ternary use of FA, MK, and cement can provide better compressive strength of UHPC than the binary use of SF and cement [55]. The 20% and 3.8% of cement were replaced with FA and MK, respectively, and the compressive strength increased by 26% at 28 days. Yazıcı et al. found that the ternary SF-FA-GGBS binder system is effective for reducing SF and water demand without sacrificing compressive strength [54]. In the binder system, the 10% replacement ratio of cement with FA increased the compressive strength by 4.1% up to 281 MPa at 1 day under the autoclave curing condition. However, a fundamental understanding of the ternary use of SCMs improving mechanical properties of UHPC is still not clear and requires further research.

Table 6 summarizes the effect of FA on the UHPC flowability, which is controversial among studies. Some researchers reported that FA can improve the workability of UHPC. Li found that the ternary use of FA, MK, and cement can significantly increase the flowability of UHPC by 47% compared to the binary use of cement and SF [55]. Randl et al. found that the addition of FA can increase the flowability of UHPC [42]. The 38.5% replacement ratio of cement with FA can increase the flow diameter of fresh UHPC by 3.6% compared to the reference specimen with SF only. Ferdosian and Camões focused on developing a mixing design method to minimize CO_2_ content and material cost with acceptable compressive strength and workability [56]. The 34.1% FA in the cementitious binder satisfied the low limit of flowability of 190 mm. However, degradation of workability by using FA in UHPC was reported. Pyo and Kim found that the 15.7% replacement of silica powder with FA decreases the slump flow by 6.6% compared to the reference specimen [40]. Ahmad et al. studied that the effect of FA replaces the SF in UHPC. It was found that when the use of FA as a replacement of SF and its content is increased to 11.8% of binder, the flowability of UHPC slightly decreases by 8.7% than the reference specimen with SF only but the lower flowability is still acceptable [49].

Table 7 summarizes the effect of FA on UHPC shrinkage. It has been shown that the FA can reduce the shrinkage of UHPC. Li et al. found that the ternary use of FA, MK, and cement can reduce the drying shrinkage of UHPC compared to the reference specimen with SF only because the ternary use can reduce water demand [55]. Yazıcı et al. replaced cement with FA and GGBS to reduce the cement amount in UHPC [54]. It was found that when the content of GGBS in the binder is constant, the 10% replacement ratio of cement with FA results in lower shrinkage than the reference specimen with SF only because of the lower amount of cement in UHPC.

The advantage of the usage of FA in UHPC cannot be observed in the compressive strength; most studies using FA reported degradation of the compressive strength of UHPC. The effect of FA on workability is arguable, and this might come from different characteristics of FAs from different sources. Therefore, the purpose of the usage of FA can be limited in reducing material cost or CO_2_ emission as discussed in the studies. Perhaps FA can increase the durability of UHPC; however, further studies are required to demonstrate it.

### 3.3. Limestone Powder (LP)

The effect of LP in conventional cement and concrete is well known; the use of LP in concrete has various advantages. It can reduce the material cost and CO_2_ emission because of having an abundant reservoir. LP has the nucleation effect in early hydration reaction that accelerates the cement hydration [58,80]. It can also physically fill the void and increase the packing density of the system [80]. As a consequence, LP increases the compressive strength of concrete at an early age. However, it may reduce the compressive strength at a late age because it does not have a pozzolanic reaction with cement unlike other SCMs such as slag, FA, and MK, and it requires higher water demand. In this section, the effect of LP in UHPC is reviewed

The effect of FA on UHPC compressive strength is summarized in Table 8. Three different mechanisms of how LP affects the compressive strength of UHPC were observed. First, LP enables the reduction in the amount of superplasticizer to maintain the same flowability. Huang et al. studied the effect of LP on the hydration of UHPC with different cement replacement ratios [58]. The retardation effect caused by the superplasticizer decreases as LP enables the reduction in the amount of superplasticizer by 62.8%, and, as a result, the early compressive strength is not degraded. It was also found that the 32% replacement ratio of cement with LP results in 10.7% and 16.1% higher compressive strength at 28 days and 56 days, respectively.

Second, LP has a pozzolanic reaction with SF. Li et al. adopted that replacing cement with LP and the optimum content of 37.3% replacement ratio increases the compressive strength of UHPC by 4.3% at 28 days than that of the control mix with SF only [57]. As UHPC with LP has a higher pozzolanic reaction with SF, which contributes to the CSH formation at late ages, strength development can be improved at late ages.

Third, the fine particle-sized LP can accelerate cement hydration. Wu et al. reported the addition of nanoparticles such as NC can improve the mechanical properties and durability of UHPC [60]. It is found that the 3.2% replacement ratio of cement with NC increases the compressive strength of UHPC by 1.1% and 5% at 7 days and 28 days, respectively, because the use of NC accelerates the hydration of cement and makes the microstructure denser due to smaller particle size of NC compared to cement.

However, in some cases, LP can degrade the compressive strength of UHPC. Yang et al. investigated the effect of LP on the hardened properties of the UHPC that contains FA and SF of 24% and 12%, respectively [59]. The 14% replacement ratio of cement with LP decreases the compressive strength of UHPC by 4.2% at 7 days than that of the reference specimen with SF and FA. Although the compressive strengths of UHPC at 28 days and 56 days increase compared to day 7 compressive strength, these two strengths are still 1.1% and 4% lower than the reference specimen, respectively. Even though the LP has the nucleation effect increasing the cement hydration speed, it also dilutes the cement hydration resulting in lower heat of cement hydration. As the amount of LP increases in the low cement binder, the dilution effect becomes more dominant. Ahmad et al. studied the effect of the use of locally available industrial waste material such as LP as a partial substitution of SF [49]. The use of LP decreases the compressive strength of UHPC by 5.65% when the content of LP increases to 4% of the binder compared to the reference specimen with SF only.

LP can significantly improve the workability of UHPC as shown in Table 9. Li et al. insisted that LP can be regarded as a mineral plasticizer that improves the flowability of the UHPC [57]. The 37.3% replacement ratio of cement with LP results in 45.1% higher flowability than that of the reference specimen that contains SF only. The plasticization effect of LP increases the workability of UHPC because of the repulsion between OH^-^ group localized on the Ca^2+^ surface and its lower water absorption [57]. Yang et al. found that the use of LP as a partial substitution of cement can enhance the flowability of UHPC [59]. The 14% replacement ratio of cement with LP increases the flow diameter by 65.5% than the reference specimen with SF only. This can be attributed to the higher w/c ratio as a part of cement is replaced with LP. Ahmad et al. also found that the use of LP increases the flowability of UHPC by 10.9% when the content of LP is 4% of the binder compared to the reference specimen with SF only [49].

LP can lower the shrinkage of UHPC by reducing the amount of cement in UHPC as shown in Table 10. Li et al. found that a 57.2% replacement ratio of cement with LP can improve the total shrinkage of UHPC compared to that of the reference specimen with SF only [57]. The study insisted that the lower amount of cement in UHPC replaced with LP slows down the hydration and reduces the hydration products, and, thus, results in the lower autogenous shrinkage. It should be pointed out, however, that the high content of LP up to 78.1% of the binder provides more free water, and, thus, drying shrinkage increases. In consequence, the total shrinkage decreases because the reduction in autogenous shrinkage is greater than the increase in drying shrinkage. Yang et al. also reported that replacing 14% cement with LP reduces the autogenous and dry shrinkage compared to the reference specimen with SF only [59].

Although three different mechanisms of how LP increases the compressive strength of UHPC have been proposed, the actual performance of LP in UHPC is debatable. From the literature, it was confirmed that LP increases the workability of UPHC. Therefore, the mechanism of LP to improve the compressive strength of UHPC by reducing water content seems appropriate. The finer LP enhances the compressive strength of UHPC by accelerating the cement hydration. Some studies insisted that the addition of LP decreases the amount of cement in UHPC, which degrades the compressive strength of UHPC. However, their dosages are lower than the other studies showing higher compressive strength with LP, and, therefore, other unknown factors of LP were assumed to degrade the compressive strength.

### 3.4. Metakaolin (MK)

MK obtained by calcining kaolin has the main chemical composition of alumina and silica, and, therefore, MK is also a pozzolanic material. Studies have reported that MK increases the durability of concrete: low permeability, high resistance against frost, and chemical attack [81,82,83].

The effect of MK on the UHPC compressive strength is summarized in Table 11. The use of MK only seems to increase the early age compressive strength of UHPC but decreases the late age compressive strength of UHPC. Li et al. found that replacing cement with MK can improve early age compressive strength but the late age compressive strength is decreased compared to UHPC with SF only [63]. It was found that the 16.7% replacement ratio of cement with MK results in 47% higher 1-day compressive strength than the reference specimen with SF only because the use of MK improves the cement hydration at an early age. However, it decreases the 28-day compressive strength by 11.8% compared to the reference sample, of which impact is less significant compared to that of 1-day compressive strength. Tafraoui et al. found a replacement of SF of 20% with MK decreasing the 28-day compressive strength of UHPC by 26.1% with steam curing, and by 5.8% with water curing, respectively [61,62]. The more significant loss of 26.1% compared to 5.8%, even with the same dosage of MK, is because of the usage of the crushed quartz that can lower the compactness by a looseness of granular stacking.

NMK may overcome the degradation of the UPHC compressive strength caused by MK. Muhd Norhasri et al. indicated that the inclusion of NMK in UHPC can achieve a similar compressive strength at early ages compared to the UHPC with MK only [64]. NMK inclusion of 1% in UHPC can increase the compressive strength of UHPC at 28 days by 7.9% than that of the reference specimen with MK only because nano-MK provides a moderate ultra-filling effect in densifying the UHPC. The disadvantage of NMK is that it decreases the workability of UPHC; 1% NMK in UHPC decreases the slump flow by 2.4% because of the higher surface of NMK than that of MK (See Table 12).

The effect of MK on the shrinkage of UHPC can be different concerning the type of shrinkage measured as shown in Table 13. Li and Rangaraju studied the effect of MK on the shrinkage of UHPC [63]. The addition of MK of 16.7% increases the autogenous shrinkage by 0.16%, but it decreases the drying shrinkage by 0.1%. However, no clear explanation of the different effects of MK on the different types of shrinkage is proposed.

MK can be incorporated in alkali-activated material (AAM). Wetzel and Middendorf introduced the UHPC made by AAM. Slag, MK, and SF were mixed with hydroxide solution and glass water [65]. The specimens were cured at 60 °C and exhibit a compressive strength at 28 days over 150 MPa. The alkalinity of AAM is higher than ordinary Portland cement; the pH of AAM is usually over 14, whereas that of ordinary Portland cement is 12.6–13.5. Due to the highly alkaline environment of AAM, SF even increases the workability of UPHC and MK reduces much less than the case of ordinary Portland cement. As a result, AAM concrete shows good workability. MK creates the geopolymer network of Si-O-T (Si, Al) in AAM which increases the chemical attack resistance of UHPC.

MK tends to decrease the compressive strength of UHPC. It decreases the workability of UHPC and its beneficial effect on the shrinkage is not clear. Based on the fact that MK is not naturally stored but needs to be calcined, it also is difficult to find the merits of MK in material cost and CO_2_ emission compared to slag, FA, or LP. Therefore, the usage of MK in UHPC seems not suitable. However, another possible application was found; the geopolymer or alkali-activated concrete resulted in a compressive strength of over 150 MPa. As geopolymer is well known for its lower CO_2_ emission compared to OPC, developing geopolymer UHPC with MK can be an interesting research subject.

### 3.5. Other SCMs

Studies adopting other SCMs that do not belong to the SCM categories of slag, FA, LP, and MK to reduce the amount of cement and SF in UPHC are summarized in this subsection. Here are the summaries of the studies reviewed in this paper. Table 14, Table 15 and Table 16 summarize the effects of other SCMs on the compressive strength, the flowability, and the shrinkage of UHPC, respectively.

#### 3.5.1. Rice Husk Ash (RHA)

RHA obtained by burning rice husk has a very high specific surface area, higher than 250 m^2^/g. The small particle size and the amorphous structure of RHA make it a “highly active pozzolan”. Van Tuan et al. indicated that cement hydration can be accelerated by the addition of RHA of which mean particle size is 5.6 μm, and it can reduce porosity and improve the compressive strength of concretes [66]. The 10% replacement ratio of cement with RHA can increase compressive strength by 10.6% and 8.8% at 3 days and 28 days, respectively. It was also found when the grinding time increases to produce the fine RHA, the pore structure of RHA is gradually collapsed resulting in the lower porosity of RHA. This collapse of RHA can improve the compressive strength of UHPC. It was also found that SF and RHA has a synergic effect on the compressive strength of UHPC; the SF contributes to the early age compressive strength, while RHA to the late age compressive strength.

#### 3.5.2. Natural Pozzolan (NP)

NP obtained from volcanic rocks is a raw material that shows pozzolanic properties so it can lower both costs and CO_2_ emission of concrete. The content of NP up to 11.8% of binder in UHPC decreases the compressive strength at 28 days by 4.3% compared to the reference specimen with SF only [67]. It was found that the replacement of 24% cement with NP decreases the compressive strength at all ages compared to the reference specimen without NP, but the compressive strength of UHPC is still over 150 MPa at 90 days. NP also decreases the flowability by 15.2% because of its higher specific surface area (6666 cm^2^/g) than that of Portland cement (3700 cm^2^/g)

#### 3.5.3. Nano-Metaclay (NMC)

Norhasri et al. adopted the NMC made from nanoclay which undergoes calcination for 3 h [68]. The particle size of NMC is very small, as much as 20 nm, which increases the water demand and retards cement hydration. The replacement of the cement of 1% with NMC decreases the compressive strength by 16.7%, 13.3%, and 3.0% compared to the reference specimen with MK only at 3 days, 7 days, and 28 days, respectively. However, the use of NMC increases the 90-day compressive strength by 6.5% because it can fill pores and yield a pozzolanic reaction at late ages. The higher surface area of NMC than that of cement and MK led to decreases in workability; the 1% inclusion of NMC in the UHPC paste reduces the slump flow of UHPC by 6.6% compared to the reference specimen.

#### 3.5.4. Dehydrated Cementitious Powder (DCP)

DCP can be obtained from recycled construction waste cementitious materials by heating up to 1000 °C. High temperature dehydrates hydrated products such as ettringite, CSH gel and Ca(OH)_2_. The dehydrated hydration product will rebuild new hydration products, which are similar to the initial hydration products. Since high temperature is essential to produce DCP from construction wastes, it may not reduce the CO_2_ emission; however, it can resolve an issue with a large amount of construction wastes. Qian et al. found that the replacement of cement up to 9% with DCP almost has no significant effect on the compressive strength of UHPC compared to the reference specimen with SF only [69]. However, DCP decreases the flowability by 18.9% because of the higher water demand caused by its larger specific surface area. It was also observed that the internal unstable CSH structure and the rehydration of CaO and other substances in DCP consume more water after heating treatment.

#### 3.5.5. Cement Kiln Dust (CKD)

Ahmad et al. studied the effect of CKD on the compressive strength of UHPC as a partial substitution of SF [49]. CKD is the fine-grained, solid, and strong alkaline waste removed from cement kiln exhaust gas by air pollution control devices in a cement plant. The content of CKD of up to 4% of the binder can obtain the compressive strength of over 150 MPa at 28 days but still 5.6% lower than that of reference specimen with SF. The addition of CKD decreased the mini-slump value by 4.3% compared to the reference mix. This is attributed to the high CaO content in CKD up to 49.3%. It is reported that the high CaO content in CKD increases the water demand.

#### 3.5.6. Ground Granite Powder (GGP)

GGP can be obtained from stone processing plants. Since GGP is an industrial waste, it can reduce the cost of UHPC by replacing parts of the SF and cement. The pore structure of the cement matrix is improved mainly because GGP is finer than cement, and it can help fill the pores in the hardened cement matrix. It was found that the replacement of 11.5% cement with GGP increases the compressive strength by 15.4% at 28 days than the reference specimen without GGP [70]. Since GGP works as a filler and does not have a pozzolanic reaction in UHPC, the GGP over an optimum amount yielded lower strength. GGP increases the workability of UHPC because GGP lowers the viscosity of the UHPC mortar as it does not react with cementitious material, resulting in increased the flowability by 4.2% compared to the reference specimen.

#### 3.5.7. Basalt Stone Powder (BP)

Yang et al. exploited a BP to reduce the cement amount in UHPC. BP is a type of stone powder obtained from aggregate and its main particle size is around 10–50 µm [59]. The replacement of 14% cement with BP results in a compressive strength of 16.7%, 1.1%, and 10.9% lower at 7, 28, and 56 days, respectively, compared to the reference specimen with SF and FA only because BP has no chemical effect in cement hydration and only plays a role as filler in UHPC. BP can improve the flowability resulting in a 58.3% higher flowability than that of the reference specimen. This can be attributed to the dilution effect of the added BP and its lower water absorption. That BP decreases the shrinkage of UHPC was also found. The BP dosage of 14% to the total binder resulted in lower autogenous and drying shrinkage because the usage of BP reduces the amount of cement in UHPC, and BP can make the microstructure denser so that the surface water evaporates slowly compared to the reference specimen with SF and FA only.

#### 3.5.8. Fine Glass Powder (FGP)

Soliman and Tagnit-Hamou found that the use of FGP as a partial substitution of SF can improve both compressive strength and workability [71]. When replacing SF with FGP up to 6% of the total binder, the 28-day compressive strength of UHPC increases by 5.0% than the reference specimen with SF only. This is attributed to the pozzolanic reaction from SF and FGP. The use of FGP also increases the workability and when the content of FGP up to 6% of the binder the slump flow increases by 18.4% compared to the reference specimen because FGP can decrease the water demand of UHPC with the lower surface area of FGP than that of SF.

RHA and FGP are suitable to improve the compressive strength of UHPC at all ages. The high pozzolanic reactivity of materials resulted in a beneficial effect on the compressive strength. GCP also enhances the compressive strength; however, it works as a filler having no chemical reactions with cement. Other SCMs introduced in this paper degrade the compressive strength, and, therefore, the purpose of their application can be considered as reducing environmental impacts.

## 4. Environmental Evaluation

The purposes of the usage of SCMs are mainly to reduce material costs and to reduce environmental burdens. Summarizing the comparison of material costs is impractical because the industrial circumstances are different between regions. Therefore, this paper provides a summary of the environmental impact data reviewed in this study.

This paper adopted the embodied carbon dioxide (e-CO_2_) and energy consumption (e-Energy) data of the raw materials provided by the previous studies [84,85,86], as shown in Table 17. The embodied CO_2_ and the embodied energy are based on the carbon footprint per unit (kg/kg) of each material and the quantity of non-renewable energy per unit (MJ/kg) of each raw material, respectively. The embodied CO_2_ of the UHPC per the unit weight of 1 kg is calculated as the sum of the values obtained by multiplying the carbon footprint values in Table 17 and the mass ratios of each raw material in Table 18; the calculation method of the e-Energy of UHPC is similar to that of embodied CO_2_ except using the e-Energy values in Table 17. However, since information is limited in the SCMs of cement, SF, FA, GGBS, MK, and LP, the other SCMs reviewed in Section 3.5 could not be analyzed. The fibers were not taken into account for the calculation because the fiber may dilute the effect of SCMs as not all of the studies applied fibers. Superplasticizers were also not included in the calculation because their dosage in UHPC is relatively very low compared to other ingredients. It is noted that different names of the SCMs are classified to a specific type of SCMs; for example, GGBS, SSP, FGGBS, LTS, SSP, etc. are considered to have the same e-CO_2_ and e-Energy values as GGBS in Table 17. Additionally, the influence of the curing method on e-CO_2_ and e-Energy was ignored. Therefore, the data provided in this study have potential errors.

**Figure 1:**
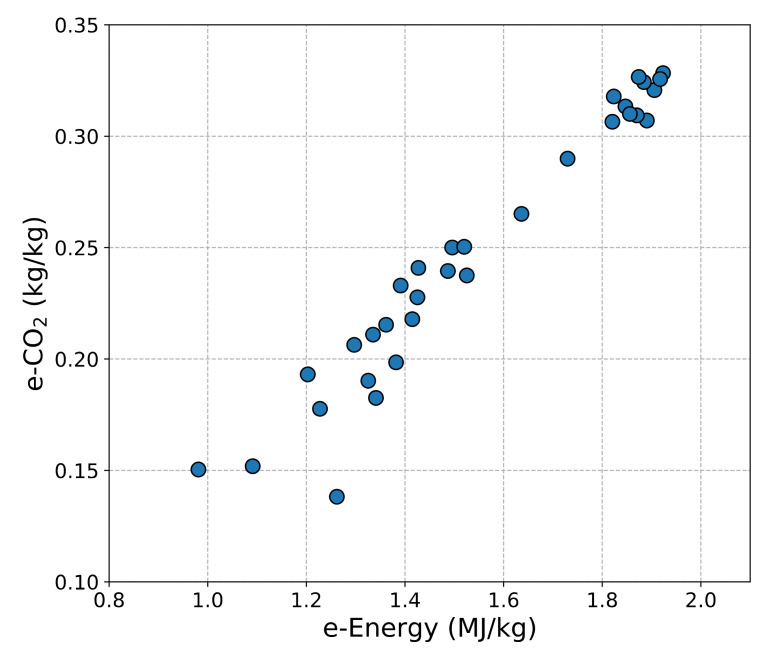
The linear relationship between e-CO_2_ and e-Energy.

**Figure 2:**
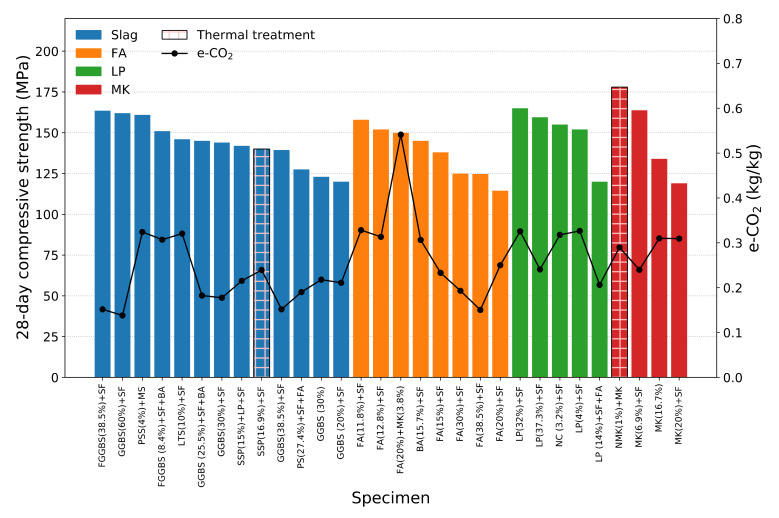
The 28-days compressive strength and the e-CO_2_ of the UHPC reviewed in this paper.

The relationship between e-CO_2_ and e-Energy is almost linear as shown in Figure 1 indicating that the energy used to produce the material also generates CO_2_ proportionally. Therefore, the e-CO_2_ data are used to investigate the environmental impact of the UHPC mix designs reviewed in this study. Figure 2 shows the summary of the e-CO_2_ and the 28-day compressive strength of the UHPCs. The bar graph corresponds to the 28-day compressive strength of the left *Y*-axis and the line plot to the e-CO_2_ data of the right *Y*-axis, and the hatched ones mean that the specimen was thermally treated. The data are divided by the type of SCMs used and, then, sorted by the 28-day compressive strength in descending order. More detailed information on the e-CO_2_ and e-Energy can also be found in Table 18. It can be concluded that the 28-day compressive strength of UHPC is not always correlated to the e-CO_2_ data. This implies the possibilities of optimizing the UHPC mix design for higher compressive strength as well as the lower e-CO_2_ of UHPC. However, the investigation on the e-CO_2_ and e-Energy of various types of SCMs should be preceded for the accurate analysis. Slag seems to have a lower environmental impact compared to other SCMs because of its higher dosage. Therefore, the applicable dosage of raw SCM material is also an important factor contributing to the decrease in the e-CO_2_ of UHPC. It is believed that the summary data can show a comprehensive understanding of which SCMs are more efficient to reduce the environmental impact and to have higher compressive strength.

## 5. Conclusions

This paper reviewed the effect of SCMs on the properties of UHPC. The various types of SCMs such as slag, FA, LP, MK, and others were successfully applied to UHPC, satisfying material requirements such as compressive strength. Based on the discussions, their effects are summarized as follows:

(1) The main purposes of the usage of SCMs are to decrease the material cost and the environmental impact caused during material production by a partial replacement of cement or silica fume. Since most SCMs are industrial by-products from plants or naturally occurring resources, the usage of SCMs corresponds well to this purpose; it was confirmed that the e-CO_2_ of UHPC is lower when the dosage of an SCM is higher.
(2) Slag tends to decrease the compressive strength of UHPC at an early age because of the slow hydration of slag, but it increases the late age compressive strength through the pozzolanic reaction between slag and Ca(OH)_2_ that increases the packing density of the UHPC. The finer particle size of slag exhibits higher compressive strength. Slag also increases the workability of UHPC because of its lower water absorption compared to cement.
(3) FA degrades the compressive strength of UHPC; however, some of the FFA can enhance compressive strength. The ternary use of SCMs including FA can be another feasible option to reduce the amount of cement in UHPC. The effect of FA on the workability of UPHC is different among studies. It is also proved that FA is effective to reduce the shrinkage of UHPC.
(4) LP enhances the compressive strength of UHPC with the three mechanisms: i) LP decreases the water demand of UHPC, that is, it increases the workability of UHPC, ii) LP has a pozzolanic reaction with SF, which increases the late age compressive strength, and iii) LP can accelerate the cement hydration. However, some cases that LP degrades the compressive strength of UHPC were observed. LP can decrease the shrinkage of UHPC by reducing the amount of cement in UHPC.
(5) MK seems to increase the early age compressive strength of UHPC, but decreases the late age compressive strength. It was confirmed that the MK of the finer particle size can overcome the degradation of the early age compressive strength. It was reported that MK decreases the autogenous shrinkage while it increases the drying shrinkage. Another application of MK was found; the alkali-activated material synthesized using slag, MK, and sodium silicate solution results in the proper compressive strength over 150 MPa.
(6) Other SCMs are also introduced. RHA has a synergic effect on the compressive strength of UHPC resulting in the higher compressive strength at both early and late age compared to the reference specimen only with SF. NP decreases the compressive strength of UHPC at all ages; however, it results in the compressive strength of UHPC over 150 MPa at 90 days. NMC increases the late age compressive strength of UHPC because it yields a pozzolanic reaction at late ages. DCP and CDK degrade the compressive strength of UHPC because they increase the water demand. GCP is a good source of SCM; it improves both the compressive strength at 28 days and the flowability of UHPC. GCP does not chemically react in UHPC but works as a filler. BP was confirmed to decrease the compressive strength of UHPC, but it increases the workability. Partial substitution of SF with FGP can improve both the compressive strength because of its pozzolanic reaction and advance the workability of UHPC because of the lower surface area compared to SF.

Although this paper examined the effect of extensively various SCMs on UHPC properties, the properties themselves are limited in compressive strength, flowability, shrinkage, and environmental impact. Due to the small number of studies found in the literature, other important properties of UHPC such as tensile strength, modulus of elasticity, and fracture energy were not able to be summarized in this paper. Therefore, further studies should be proceeded for a review of the effect of SCMs on those properties.

## Figures and Tables

**Table 1:** Summary of the effects of supplementary cementitious materials (SCMs) on the UHPC performances.

Performance		SCM No.	List of SCMs	
Early compressive strength (≤3 days)	High	[Slag] 7, 13; [FA] 23, 25; [MK] 32; [O] 35	**[Slag]** (1) **GGBS (60%)** + SF [39] (2) **GGBS (25.5%)** + SF + BA [40] (3) **GGBS (30%)** + SF [41] (4) **GGBS (38.5%)** + SF [42] (5) *GGBS (30%)* [43] (6) **GGBS (20–40%)** + SF [44] (7) **GGBS (23.6%)** + SF [45] (8) **FGGBS (38.5%)** + SF [42] (9) **FGGBS (8.4%)** + SF + BA [40] (10) **SSP (16.9%)** + SF [46] (11) **SSP (15%)** + LP + SF [12] (12) **PS (6.9–34.2%)** + SF + FA [47] (13) **PS (35%)** + SF [48] (14) **PSS (4%)** + SF [49] (15) **LTS (10%)** + SF [50] (16) **CS (16%)** + SF [51] **Fly ash [FA]** (17) **FA (11.8%)** + SF [49] (18) **FA (12.8%)** + SF [40] (19) **FA (15%)** + SF [41] (20) **FA (30%)** + SF [52] (21) **FA (20%)** + SF [53] (22) **FA (38.5%)** + SF [42] (23) **FA (7.4%)** + GGBS + SF [54] (24) *FFA (20%) + MK (3.8%)* [55] (25) **FFA (34.1%)** + SF [56]	**Limestone powder [LP]** (26-1) **LP (37.3%)** + SF [57] (26-2) **LP (57.2–78.1%)** + SF [57] (27) **LP (32%)** + SF [58] (28) **LP (4%)** + SF [49] (29) **LP (14%)** + SF + FA [59] (30) **NC (3.2%)** + SF [60] **Metakaolin [MK]** (31) **MK (20%)** + SF [61,62] (32) *MK (16.7%)* [63] (33) **NMK (1%)** + MK [64] (34) **MK (6.9%)** + SF [65] **Others [O]** (35) **RHA (10%)** + SF [66] (36) **NP (11.8%)** + SF [49] (37) **NP (24%)** + SF [67] (38) **NMC (1–9%)** + MK [68] (39) **DCP (≤9%)** + SF + LP [69] (40) **CKD (4%)** + SF [49] (41) **GGP (11.5%)** + SF [70] (42) **FGP (6–15%)** + SF [71] (43) **BP (14%)** + SF + FA [59]
	Low	[Slag] 1, 2, 3, 9, 11, 12, 15; [FA] 18, 19, 21; [LP] 29; [MK] 33; [O] 37, 38, 39, 43		
Late compressive strength (>3 days)	High	[Slag] 1, 2, 5, 6, 7, 9, 12, 14, 15, 16; [FA] 20, 24; [LP] 26-1, 27, 30; [MK] 32; [O] 35, 41, 42		
	Low	[Slag] 3, 4, 8, 10, 11; [FA] 17, 18, 19, 21, 22; [LP] 26-2, 28, 29; [MK] 31, 34, 32; [O] 36, 37, 38, 39, 40, 43		
Flowability	High	[Slag] 2, 4, 6, 7, 8, 9, 10, 12; [FA] 22, 24, 25; [LP] 26-1, 26-2, 28, 29; [O] 41, 42, 43		
	Low	[Slag] 11; [FA] 17, 18; [MK] 33; [O] 36, 37, 38, 39, 40		
Shrinkage	Low	[Slag] 11, 12; [FA] 23, 24; [LP] 26-2, 29; [MK] 32; [O] 43		
	High	[Slag] 6		

**Table 2:** The effect of slag on the compressive strength of UHPC.

SCMs	Compressive Strength (MPa @ Age (% to the Ref.))	w/b Ratio	Curing Method	Specimen Size (mm)	Other Solid Ingredients	Ref.
**GGBS (60%)** + SF	127 @ 7 (−7.1%) 162 @ 28 (5.2%) 181 @ 90 (8.4%)	0.20	Water	50 cube	Cement (CEM I 52.5 N), Sand	[39]
**GGBS (25.5%)** + SF + BA	25 @ 1 (−39.0%) 77 @ 3 (−18.1%) 145 @ 28 (0%) 157 @ 91 (0.06%)	0.15	Water	50 cube	Cement (CEM I), Sand, Steel fiber (1 vol.%), Silica powder	[40]
**GGBS (30%)** + SF	98 @ 3 (−5.7%) 144 @ 28 (−4.0%)	0.16	Water	40 × 40 × 80	Cement (CEM I 42.5 R), Sand, Steel fiber (2 vol.%)	[41]
**GGBS (38.5%)** + SF	139.4 @ 28 (−16.1%)	0.20	Water and air	100 cube	Cement (CEM I 42.5), Sand, Steel fiber (2 vol.%)	[42]
***GGBS (30%)***	123 @ 28 (10%) 130 @ 91 (−5.4%)	0.18	Water	40 × 40 × 160	Cement (CEM I 52.5 R), Sand	[43]
**GGBS (20–40%)** +SF	110–120 @ 28 (0–9%)	0.18	Water	50 cube	Cement (CEM I), Sand	[44]
**GGBS (23.6%)** + SF	110 @ 3 (0.0%) 125 @ 7 (3.3%)	0.14	Air	40 × 40 × 160	Cement (CEM I 52.5 N), Sand	[45]
**FGGBS (38.5%)** + SF	163.5 @ 28 (−1.5%)	0.20	Water and air	100 cube	Cement (CEM I 42.5 R), Sand, Steel fiber (2 vol.%)	[42]
**FGGBS (8.4%)** + SF + BA	13 @ 1 (−68.3%) 101 @ 3 (7.4%) 151 @ 28 (4.1%) 165 @ 91 (5.8%)	0.15	Water	50 cube	Cement (CEM I), Sand, Steel fiber (1 vol.%), Silica powder	[40]
**SSP (16.9%)** + SF	140 @ 28 (−10.3%)	0.13	Heat, water	100 cube	Cement (CEM I 42.5), Sand, Coarse agg., Steel fiber (1.6 vol.%)	[46]
**SSP (15%)** + LP + SF	68 @ 1 (−8.7%) 142 @ 28 (−6.4%)	0.16	Water	100 cube	Cement (CEM I 42.5), Sand, Quartz powder, Steel fiber (2 vol.%)	[12]
**PS (27.4%)** + SF + FA	60 @ 3 (−27.7%) 127.5 @ 28 (2.8%)	0.17	Air	40 × 40 × 160	Cement (CEM I), Sand	[47]
**PS (35%)** + SF	156.8 @ 3 (3.7%)	0.14	Heat	40 × 40 × 160	Cement (CEM I 52.5), Sand	[48]
**PSS (4%)** + SF	161 @ 28 (0.0%)	0.15	Water		Cement (CEM I), Sand, Steel fiber (2 vol.%)	[49]
**LTS (10%)** + SF	98 @ 3 (−4.8%) 146 @ 28 (2.8%) 156 @ 90 (6.8%)	0.18	Water	40 cube	Cement (CEM I 52.5), Sand	[50]
**CS (16%)** + SF	167 @ 90 (3.1%)	0.15	Water	40 × 40 × 160	Cement (CEM I 52.5 N), Sand	[51]

**Table 3:** Effect of slag on the flowability of UHPC.

SCMs	Flowability (mm (% to the Ref.))			w/b Ratio	SP/b Ratio	Agg/b Ratio	Type	Ref.
	Slump Flow	Flow Table	Mini Slump					
**FGGBS (8.4%)** + SF + BA	675 (11.6%)			0.15	0.75%	0.70	Mortar + Steel fiber (1 vol.%)	[40]
**GGBS (25.5%)** + SF + BA	630 (4.1%)			0.15	0.49%	0.70	Mortar + Steel fiber (1 vol.%)	[40]
**SSP (15%)** + LP + SF	605 (−0.1%)			0.16	1.80%	1.00	Mortar + Steel fiber (2 vol.%)	[12]
**PS (34.2%)** + SF + FA		306 (17.2%)		0.17	3.47%	0.90	Mortar	[47]
**FGGBS (38.5%)** + SF	310 (10.7%)			0.20	3.50%	1.44	Mortar + Steel fiber (2 vol.%)	[42]
**GGBS (23.6%)** + SF			300 (0.0%)	0.14	0.90%	1.00	Mortar	[45]
**GGBS (38.5%)** + SF	285 (1.8%)			0.20	3.50%	1.44	Mortar + Steel fiber (2 vol.%)	[42]
**GGBS (20–40%)** + SF			256 (34.7%)	0.18	2.40%	1.22	Mortar	[44]
**SSP (16.9%)** + SF		130 (26.8%)		0.13	5.42%	1.25	Mortar	[46]

**Table 4:** Effect of slag on the shrinkage of UHPC.

SCMs	Shrinkage			w/b Ratio	Binder Weight Ratio			Ref.
	Auto	Dry	Total		Cement	Slag	SF	
**SSP (15%)** + LP + SF			Low	0.16	0.55	0.35	0.10	[12]
**PS (34.2%)** + SF + FA	Low			0.17	0.34	0.53	0.13	[47]
**GGBS (40%)** + SF	High			0.18	0.40	0.40	0.20	[44]

**Table 5:** The effect of FA on the compressive strength of UHPC.

SCMs	Compressive Strength (MPa @ Age (% to the Ref.))	w/b Ratio	Curing Method	Specimen Size (mm)	Other Solid Ingredients	Ref.
**FA (11.8%)** + SF	158 @ 28 (−1.9%)	0.15	Water	50 cube	Cement (CEM I), Sand, Steel fiber (2 vol.%)	[49]
**FA (12.8%)** + SF	24 @ 1 (−48.9%) 92 @ 3 (−6.1%) 152 @ 28 (−1.3%) 164 @ 91 (0%)	0.15	Water	50 cube	Cement (CEM I), Sand, Steel fiber (1 vol.%)	[40]
**FA (15%)** + SF	90 @ 3 (−13.5%) 138 @ 28 (−8%)	0.20	Water	40 × 40 × 80	Cement (CEM I 42.5), Sand	[41]
**FA (30%)** + SF	125 @ 28 (19%)	0.26	Air	100 cube	Cement (CEM I), Sand, Coarse agg.	[52]
**FA (20%)** + SF	53.1 @ 1 (−33.7%) 101.5 @ 7 (−1.26%) 114.5 @ 28 (−0.7%) 131.7 @ 56 (2.1%) 152.1 @ 90 (−1.9%)	0.16	Water	50 cube	Cement (CEM I), Sand, Steel fiber (3 vol.%)	[53]
**FA (38.5%)** + SF	124.7 @ 28 (−24.9%)	0.20	Water and air	100 cube	Cement (CEM I 42.5 R), Sand, Steel fiber (2 vol.%)	[42]
**FA (7.4%)** + GGBS + SF	281 @ 1 (4.1%)	0.15	Autoclave	50 cube	Cement (CEM I 42.5), Sand	[54]
**FFA (20%)** + MK (3.8%)	150 @ 28 (26%)	0.20	Water	50 cube	Cement (CEM III)	[55]
**FFA (34.1%)** + SF	160.3 @ 3 (6.8%)	0.16	Water and steam	50 cube	Cement (CEM I 42.5 R), Sand, Steel fiber (1 vol.%)	[56]

**Table 6:** The effect of FA on the flowability of UHPC.

SCMs	Flowability (mm (% to the Ref.))			w/b Ratio	SP/b Ratio	Agg/b Ratio	Type	Ref.
	Slump Flow	Flow Table	Mini Slump					
**FA (12.8%)** + SF	565 (−6.6%)			0.15	0.75%	0.71	Mortar + Steel fiber (1 vol.%)	[40]
**FA (38.5%)** + SF	290 (3.6%)			0.20	3.50%	1.44	Mortar + Steel fiber (2 vol.%)	[42]
**FFA (20%)** + MK (3.8%)		258 (47%)		0.20	1.00%	-	Paste	[55]
**FA (11.8%)** + SF			210 (−8.7%)	0.15	3.57%	0.90	Mortar + Steel fiber (2 vol.%)	[49]
**FFA (34.1%)** + SF		190 (0.0%)		0.16	2.50%	1.07	Mortar + Steel fiber (1 vol.%)	[56]

**Table 7:** The effect of FA on the shrinkage of UHPC.

SCMs	Shrinkage			w/b Ratio	Binder Weight Ratio			Ref.
	Auto	Dry	Total		Cement	FA	SF	
**FFA (20%)** + MK(3.8%)		Low		0.20	0.77	0.23	-	[55]
**FA (8%)** + GGBS + SF			Low	0.15	0.64	0.16	0.20	[54]

**Table 8:** The effect of LP on the compressive strength of UHPC.

SCMs	Compressive Strength (MPa @ Age (% to the Ref.))	w/b Ratio	Curing Method	Specimen Size (mm)	Other Solid Ingredients	Ref.
**LP (37.3%)** + SF	159.5 @ 28 (4.3%)	0.20	Water	50 cube	Cement (CEM I 52.5 R), Sand	[57]
**LP (32%)** + SF	165 @ 28 (10.7%) 180 @ 56 (16.1%)	0.13	Sealed	40 × 40 × 160	Cement (CEM I 52.5 N), Sand	[58]
**LP (4%)** + SF	152 @ 28 (−5.6%)	0.15	Water	50 cube	Cement (CEM I), Sand, Steel fiber (2 vol.%)	[49]
**LP (14%)** + SF + FA	100 @ 7 (−4.2%) 120 @ 28 (−1.1%) 140 @ 56 (−4%)	0.16	Water	40 × 40 × 160	Cement (CEM I), Sand	[59]
**NC (3.2%)** + SF	120 @ 7 (9%) 155 @ 28 (15%)	0.16	Water	40 × 40 × 160	Cement (CEM I 42.5), Sand, Steel fiber (2 vol.%)	[60]

**Table 9:** The effect of LP on the flowability of UHPC.

SCMs	Flowability (mm (% to the Ref.))			w/b Ratio	SP/B Ratio	Agg/b Ratio	Type	Ref.
	Slump Flow	Flow Table	Mini Slump					
**LP (4%)** + SF			255 (10.9%)	0.15	3.57%	0.90	Mortar + Steel fiber (2 vol.%)	[49]
**LP (14%)** + SF + FA		240 (65.5%)		0.16	2.20%	0.85	Mortar	[59]
**LP (37.3%)** + SF			450 (45.1%)	0.20	1.30%	0.78	Mortar	[57]

**Table 10:** The effect of LP on the shrinkage of UHPC.

SCMs	Shrinkage			w/b Ratio	Binder Weight Ratio			Ref.
	Auto	Dry	Total		Cement	LP	SF	
**LP (57.2)** + SF			Low	0.20	0.39	0.57	0.04	[57]
**LP (14%)** + SF + FA	Low	Low	Low	0.16	0.49	0.39	0.12	[59]

**Table 11:** The effect of MK on the compressive strength of UHPC.

SCMs	Compressive Strength (MPa @ Age (% to the Ref.))	w/b Ratio	Curing Method	Specimen Size (mm)	Other Solid Ingredients	Ref.
**MK (20%)** + SF	119, 178, 183 @ 28 (−26.1%, 8.7%, −13.7%) (23 °C, 90 °C, 150 °C)	0.22	Water at 23 °C; and steam at 90 and 150 °C	40 × 40 × 160	Cement (CEM I 42.5), Sand	[61]
**MK (20%)** + SF	146 @ 28 (−5.8%)	0.22	Water	40 × 40 × 160	Cement (CEM I 52.5 N), Sand	[62]
**MK (16.7%)**	106 @ 3 (47.0%) 134 @ 28 (−11.8%)	0.20	Water	50 cube	Cement (CEM III), Sand	[63]
**NMK (1%)** + MK	120 @ 3 (−0.8%) 146 @ 7 (−1.3%) 178 @ 28 (7.9%)	0.20	Heat	100 cube	Cement (CEM I), Sand, Coarse agg.	[64]
**MK (6.9%)** + SF	163.8 @ 28 (9.3%)	0.25	Sealed	50 cube	GGBS, SF, Potassium, Sand (Alkali-activated material)	[65]

**Table 12:** The effect of MK on the flowability of UHPC.

SCMs	Flowability (mm (% to the Ref.))			w/b Ratio	SP/b Ratio	Agg/b Ratio	Type	Ref.
	Slump Flow	Flow Table	Mini Slump					
**NMK (1%)** + MK	162 (−2.4%)			0.20	2.00%	1.00	Mortar	[64]

**Table 13:** The effect of MK on the shrinkage of UHPC.

SCMs	Shrinkage			w/b Ratio	Binder Weight Ratio			Ref.
	Auto	Dry	Total		Cement	MK	SF	
**MK (16.7%)**	High	Low		0.20	0.83	0.17	-	[63]

**Table 14:** The effect of other SCMs on the compressive strength of UHPC.

SCMs	Compressive Strength (MPa @ Age (% to the Ref.))	w/b Ratio	Curing Method	Specimen Size (mm)	Other Solid Ingredients	Ref.
**RHA (10%)** + SF	135 @ 3 (10.6%) 155 @ 7 (5.3%) 185 @ 28 (8.8%) 205 @ 91 (4.1%)	0.18	Moisture	40 cube	Cement (CEM I 52.5 N), Sand	[66]
**NP (11.8%)** + S	152 @ 28 (−4.3%)	0.15	Water	50 cube	Cement (CEM I), Sand, Steel fiber (2 vol.%)	[49]
**NP (24%)** + SF	110 @ 7 (−11.4%) 124.5 @ 14 (−6.3%) 130.6 @ 28 (−8.7%) 151 @ 90 (−6.6%)	0.15	Water	100 cube	Cement (CEM I), Sand, Steel fiber (2 vol.%)	[67]
**NMC (1–9%)** + MK	100 @ 3 (−16.7%) 130 @ 7 (−13.3%) 160 @ 28 (−3.0%) 179 @ 90 (6.5%)	0.20	Heat	100 cube	Cement (CEM I), Sand, Coarse agg.	[68]
**DCP (≤ 9%)** + SF + LP	45 @ 3 (−0.8%) 65 @ 7 (−0.3%) 100 @ 28 (−0.6%)	0.18	Water	40 × 40 × 160	Cement (CEM I 52.5), Sand	[69]
**CKD (4%)** + SF	154 @ 28 (−5.6%)	0.15	Water	50 cube	Cement (CEM I), Sand, Steel fiber (2 vol.%)	[49]
**GGP (11.5%)** +SF	188 @ 28 (15.4%)	0.18	Autoclave	40 × 40 × 160	Cement (CEM I 42.5 R), Sand, Steel fiber (2 vol.%)	[70]
**FGP (6%)** +SF	125 @ 7 (7.1%) 175 @ 28 (5.0%) 183 @ 56 (4.8%) 196 @ 91 (7.7%)	0.19	Sealed	50 cube	Cement (CEM HS), Sand, Quartz powder	[71]
**BP (14%)** + SF + FA	90 @ 7 (−16.7%) 120 @ 28 (−1.1%) 130 @ 56 (−10.9%)	0.16	Water	40 × 40 × 160	Cement (CEM I), Sand	[59]

**Table 15:** The effect of other SCMs on the flowability of UHPC.

SCMs	Flowability (mm (% to the Ref.))			w/b Ratio	SP/b Ratio	Agg/b Ratio	Type	Ref.
	Slump Flow	Flow Table	Mini Slump					
**DCP (9%)** + SF + LP		255 (−18.9%)		0.18	3.00%	0.90	Mortar	[69]
**BP (14%)** + SF + FA		230 (58.6%)		0.16	2.20%	0.85	Mortar	[59]
**FGP (6%)** +SF			225 (18.4%)	0.19	1.25%	1.13	Mortar	[71]
**CKD (4%)** + SF			220 (−4.3%)	0.15	3.57%	0.90	Mortar + Steel fiber (2 vol.%)	[49]
**GGP (11.5%)** + SF		200 (4.2%)		0.18	1.90%	1.18	Mortar + Steel fiber (2 vol.%)	[70]
**NP (11.8%)** + SF			195 (−15.2%)	0.15	3.57%	0.90	Mortar + Steel fiber (2 vol.%)	[49]
**NP (24%)** + SF		184 (−12.4%)		0.15	3.57%	0.89	Mortar + Steel fiber (2 vol.%)	[67]
**NMC (1%)** + MK	155 (−6.6%)			0.20	1.00%	1.53	Concrete	[68]

**Table 16:** The effect of other SCMs on the shrinkage of UHPC.

SCMs	Shrinkage			w/b Ratio	Binder Weight Ratio			Ref.
	Auto	Dry	Total		Cement	SCMs	SF	
**BP (14%)** + SF + FA	Low	Low		0.16	0.49	0.39	0.12	[59]

**Table 17:** The embodied carbon dioxide and energy consumption of the raw materials [84,85,86].

Items	e-CO_2_ (kg/kg)	e-Energy (MJ/kg)
Cement [85]	0.8300	4.7270
Water [85]	0.0003	0.0060
River sand [85]	0.0010	0.0220
Crushed stone [85]	0.0070	0.1130
Slag [85]	0.0190	1.5880
Fly ash [85]	0.0090	0.8330
Limestone powder [85]	0.0170	0.3500
Metakaolin [85]	0.4000	3.4800
Silica fume [84]	0.0140	0.1000
Sodium silicate [86]	1.5140	18.3000

**Table 18:** The summary of the e-CO_2_ and the e-Energy of the UHPC reviewed in this study.

Category	Binder Mix Design	Water (wt.%)	Binder (wt.%)						Aggregate (wt.%)		e-CO_2_ (kg/kg)	e-Energy (MJ/m^3^)	Ref.
			Cement	Slag	FA	LP	MK	SF	Fine	Coarse			
Slag	LTS (10%) + SF	9	38	5	0	0	0	5	43	0	0.321	1.906	[50]
	PSS (4%) + MS	7	39	2	0	0	0	8	44	0	0.324	1.885	[49]
	GGBS (30%)	7	26	11	0	0	0	0	56	0	0.218	1.415	[43]
	SSP (16.9%) + SF	5	28	7	0	0	0	7	39	13	0.239	1.487	[46]
	FGGBS (8.4%) + SF + BA	8	37	5	9	0	0	5	37	0	0.307	1.890	[40]
	CS (16%) + SF	7	32	8	0	0	0	10	43	0	0.265	1.636	[51]
	GGBS (23.6%) + SF	7	28	11	0	0	0	8	47	0	0.238	1.525	[45]
	GGBS (20%) + SF	7	25	8	0	0	0	8	51	0	0.211	1.335	[44]
	SSP (15%) + LP + SF	7	25	7	0	9	0	5	46	0	0.215	1.362	[12]
	PS (35%) + SF	7	23	16	0	0	0	7	47	0	0.199	1.382	[48]
	GGBS (38.5%) + SF	8	18	15	0	0	0	5	55	0	0.152	1.091	[42]
	FGGBS (38.5%) + SF	8	18	15	0	0	0	5	55	0	0.152	1.091	[42]
	GGBS (30%) + SF	7	21	14	0	0	0	12	46	0	0.178	1.228	[41]
	GGBS (25.5%) + SF + BA	8	21	15	9	0	0	9	38	0	0.183	1.341	[40]
	PS (27.4%) + SF + FA	8	22	11	9	0	0	6	44	0	0.190	1.325	[47]
	GGBS (60%) + SF	11	16	32	0	0	0	5	37	0	0.138	1.262	[39]
FA	FA (38.5%) + SF	8	18	0	15	0	0	5	55	0	0.150	0.981	[42]
	BA (15.7%) + SF	8	37	0	9	0	0	9	38	0	0.306	1.821	[40]
	FA (12.8%) + SF	8	37	0	7	0	0	9	38	0	0.313	1.847	[40]
	FFA (34.1%) + SF	7	27	0	15	0	0	2	48	0	0.228	1.425	[56]
	FA (20%) + SF	8	30	0	8	0	0	2	52	0	0.250	1.496	[53]
	FA (20%) + MK (3.8%)	17	64	0	17	0	3	0	0	0	0.541	3.252	[55]
	FA (30%) + SF	8	23	0	10	0	0	0	30	29	0.193	1.203	[52]
	FA (11.8%) + SF	7	39	0	6	0	0	4	44	0	0.328	1.923	[49]
	FA (7.4%) + GGBS + SF	7	30	4	4	0	0	9	47	0	0.250	1.520	[54]
	FA (15%) + SF	8	28	0	7	0	0	12	46	0	0.233	1.391	[41]
LP	LP (32%) + SF	9	39	0	0	22	0	9	21	0	0.326	1.917	[58]
	NC (3.2%) + SF	8	38	0	0	2	0	10	42	0	0.318	1.823	[60]
	LP (37.3%) + SF	10	29	0	0	19	0	3	39	0	0.241	1.427	[57]
	LP (14%) + SF + FA	8	24	0	12	7	0	6	42	0	0.206	1.297	[59]
	LP (4%) + SF	7	39	0	0	2	0	8	44	0	0.327	1.874	[49]
MK	NMK (1%) + MK	7	33	0	0	0	4	0	20	36	0.290	1.730	[64]
	MK (20%) + SF	9	33	0	0	0	8	0	49	0	0.309	1.870	[61]
	MK (16.7%)	8	34	0	0	0	7	0	51	0	0.310	1.856	[63]
	MK (6.9%) + SF ^(1)^	10	0	21	0	0	2	2	50	0	0.240	3.162	[65]

The values of water, solid binder, and aggregate are the mass ratio. ^(1)^ The specimen is an alkali-activated material and its mix design was deduced based on the mixing ratio described in the paper. It was assumed that the mass ratio of sodium silicate used is approximately 0.15, and that the mass ratio of fine aggregate is 0.50.

## Data Availability

The data presented in this study are available on request from the corresponding author.

## Abbreviations

BA	Bottom Ash
BP	Basalt stone Powder
CKD	Cement Kiln Dust
CS	Copper Slag
DCP	Dehydrated Cementitious Powder
FA	Fly Ash
FFA	Fine Fly Ash
FGGBS	Fine Ground Granulated Blast-furnace Slag
FGP	Fine Glass Powder
GGBS	Ground Granulated Blast-furnace Slag
GGP	Ground Granite Powder
LP	Limestone Powder
LTS	Lithium Slag
MK	Metakaolin
NC	Nano Calcium carbonate
NMC	Nano Metaclay
NMK	Nano Metakaolin
NP	Natural Pozzolan
OPC	Ordinary Portland Cement
PS	Phosphorous Slag
PSS	Pulverized Steel Slag
RHA	Rice Husk Ash
SF	Silica Fume
SSP	Steel Slag Powder
